# Utility of RAND/UCLA appropriateness method in validating multiple-choice questions on ECG

**DOI:** 10.1186/s12909-024-05446-7

**Published:** 2024-04-24

**Authors:** Tomohiro Kaga, Shinji Inaba, Yukari Shikano, Yasuyuki Watanabe, Tomoki Fujisawa, Yusuke Akazawa, Muneaki Ohshita, Hiroshi Kawakami, Haruhiko Higashi, Jun Aono, Takayuki Nagai, Mohammad Zahidul Islam, Muhammad Wannous, Masatsugu Sakata, Kazumichi Yamamoto, Toshi A Furukawa, Osamu Yamaguchi

**Affiliations:** 1 Ehime University, Toon, Japan; 2 Department of Cardiology, Pulmonology, Hypertension and Nephrology, Ehime University Graduate School of Medicine, Toon, Ehime 791-0295 Japan; 3 Ehime University Graduate School of Medicine, Toon, Japan; 4 Imabari City Fire Department, Imabari, Japan; 5 Department of Emergency and Critical Care Medicine, Graduate School of Medicine, Ehime University, Toon, Japan; 6 Department of Information Communication Technology ICT Division, Government of Bangladesh, Dhaka, Bangladesh; 7 Department of Computer Information Science, Higher Colleges of Technology, Abu Dhabi, UAE; 8 Departments of Health Promotion and Human Behavior, Kyoto University Graduate School of Medicine/School of Public Health, Kyoto, Japan; 9 Institute for Airway Disease, Hyogo, Japan

**Keywords:** Electrocardiogram, Multiple-choice questions, Medical education, RAND/UCLA appropriateness method

## Abstract

**Objectives:**

This study aimed to investigate the utility of the RAND/UCLA appropriateness method (RAM) in validating expert consensus-based multiple-choice questions (MCQs) on electrocardiogram (ECG).

**Methods:**

According to the RAM user’s manual, nine panelists comprising various experts who routinely handle ECGs were asked to reach a consensus in three phases: a preparatory phase (round 0), an online test phase (round 1), and a face-to-face expert panel meeting (round 2). In round 0, the objectives and future timeline of the study were elucidated to the nine expert panelists with a summary of relevant literature. In round 1, 100 ECG questions prepared by two skilled cardiologists were answered, and the success rate was calculated by dividing the number of correct answers by 9. Furthermore, the questions were stratified into “Appropriate,” “Discussion,” or “Inappropriate” according to the median score and interquartile range (IQR) of appropriateness rating by nine panelists. In round 2, the validity of the 100 ECG questions was discussed in an expert panel meeting according to the results of round 1 and finally reassessed as “Appropriate,” “Candidate,” “Revision,” and “Defer.”

**Results:**

In round 1 results, the average success rate of the nine experts was 0.89. Using the median score and IQR, 54 questions were classified as “ Discussion.” In the expert panel meeting in round 2, 23% of the original 100 questions was ultimately deemed inappropriate, although they had been prepared by two skilled cardiologists. Most of the 46 questions categorized as “Appropriate” using the median score and IQR in round 1 were considered “Appropriate” even after round 2 (44/46, 95.7%).

**Conclusions:**

The use of the median score and IQR allowed for a more objective determination of question validity. The RAM may help select appropriate questions, contributing to the preparation of higher-quality tests.

## Introduction

Globally, the electrocardiogram (ECG), including the 12-lead ECG, monitored ECG, Holter ECG, and exercise stress ECG, is a simple, noninvasive test widely employed in clinical practice [1]. ECG is useful particularly in emergency cardiovascular diseases, such as acute myocardial infarction, and an immediate management according to accurate ECG reading is essential for improving the patient’s prognosis [2, 3]. However, reading ECGs requires knowledge and training, and medical students and residents lack competency in interpreting the ECG [4, 5]. This could be attributed to numerous reasons, including the complexity of the ECG, paucity of a standardized training system, and moreover, the lack of an established method for evaluating competency in ECG interpretation [4, 6, 7].

A systematic review of ECG education elucidated that tests to evaluate ECG interpretation competency often requires validation through scientific evidence and remain challenging owing to a small number of questions and their low quality [8, 9]. In creating tests for medical education, multiple-choice questions (MCQs) are commonly used to assess competence [10]. However, several papers have demonstrated that the MCQs warrant improvement, owing to the biases of the creator of the test and other factors [11–13].

To date, there is no objective and established method for validating MCQs, although there is guidance on how to prepare MCQs. The Delphi method is one of the gold standards of consensus methods and is used worldwide in all fields, not just in medicine. A RAND/UCLA Appropriateness Method (RAM) is a modified Delphi method in the mid-1980s by the RAND Institute/University of California, Los Angeles. RAM’s advantage over the original Delphi method is that it provides higher-quality answers and an avenue for discussion rounds among experts. RAM includes face-to-face evaluation rounds, and expert meetings provide an opportunity to reflect on one’s own judgment [14]. RAM was initially developed to reach a consensus regarding a medical intervention. However, it is increasingly used as a consensus-building method in assessment system generation.

Therefore, developing a world-standard training system to assess and improve ECG interpretation skills was initiated as a project. The protocol paper on this project has already been reported [15]. This study is the first phase of this project. Thus, this study aimed to verify the feasibility and utility of the RAM in validating ECG MCQs and creating a 50-question validated test set for the next phase of our project.

## Materials and methods

**Fig. 1:**
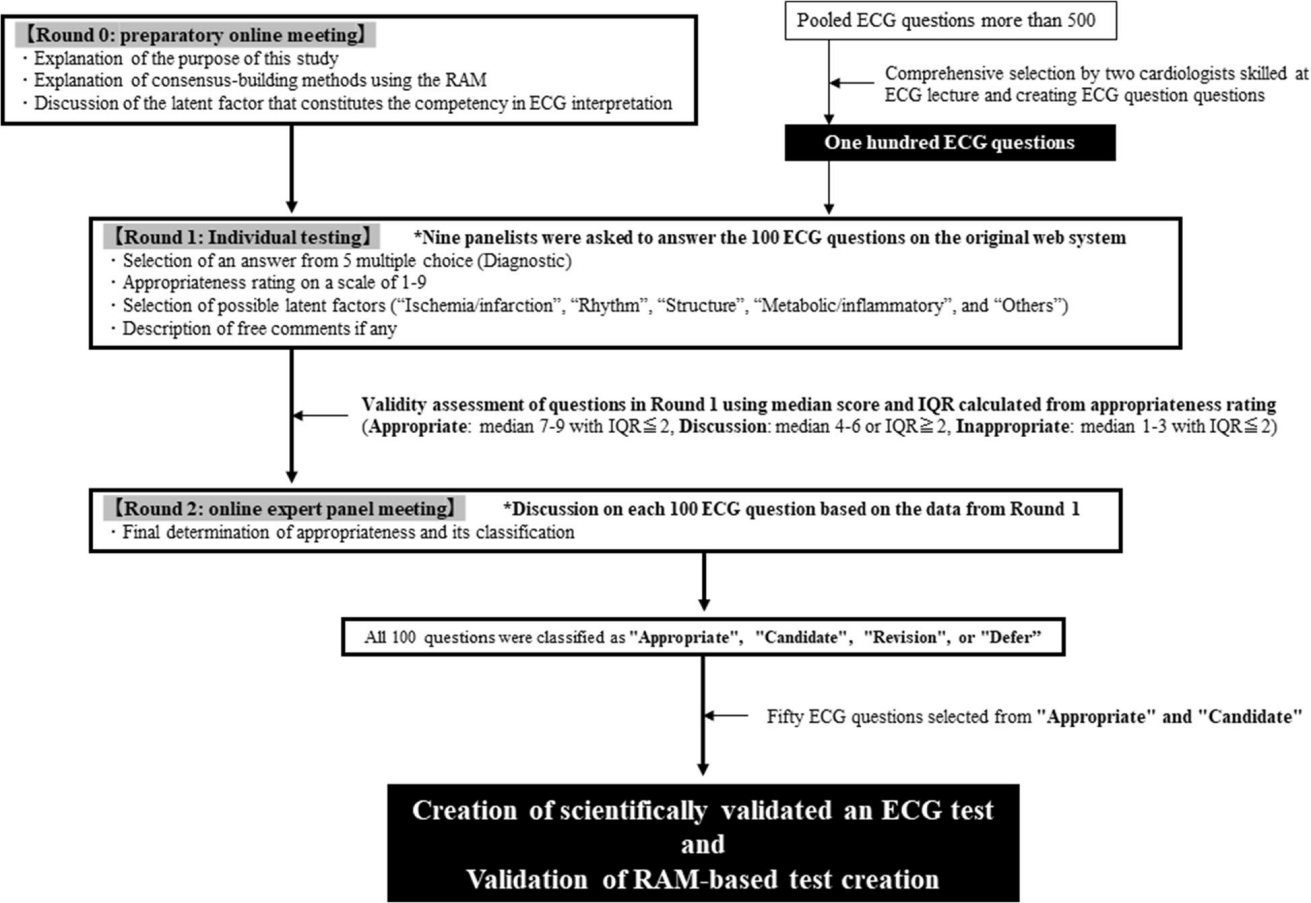
Study flowchart: Extraction of appropriate ECG questions with three rounds according to RAM. ECG = electrocardiogram; IQR = interquartile range; RAM = RAND/UCLA Appropriateness Method; MCQs = multiple-choice questions

This study was conducted from February 2023 to August 2023 and was employed according to the RAM user’s manual [14]. The details can be found in the protocol article [15]. Figure 1 briefly presents the study’s methodology. This study was approved by the research ethics committee of Ehime University Graduate School of Medicine (IRB number 2209008).

The study comprised three “rounds” ranging from 0 to 2, executed according to the sequence presented in Fig. 1. Prior to the initiation of the rounds, nine expert panelists were recruited who work with ECG in daily practice or as specialists. Our project aimed to develop highly skilled ECG professionals. Thus, ECG experts were selected from a wide range of occupations, including medical students, who are among the main targets in our project. The goal of the three RAM-based rounds is to create a scientifically validated test set through a selection of 50 questions from 100 ECG questions. Round 0 was conducted through an online meeting, and panelists who could not attend were invited to watch a meeting recording to summarize all the expert’s opinions.

In round 0, the study purpose was explained together with a summary of relevant literature and a schedule for the future. In round 1, the participants were asked to answer 100 ECG questions prepared by two cardiologists (SI and JA) skilled in ECG lectures, and questions were prepared via an online system developed for this study. The 100 ECG questions were selected from a pool of over 500 questions. Because these questions were pooled for online lectures on ECG, the quality was not ensured. Furthermore, there was also a bias in the field of questions. Therefore, to avoid bias, the first 100 ECG questions were selected by two skilled experts with reference to the Minnesota Code to ensure ECG question selection from a wide range of fields. In addition to responding to the choices, nine panelists were requested to select a category for each ECG question from the following choices: “Ischemia/infarction,” “Rhythms,” “Metabolic/inflammatory,” “Structural,” and “Others.” The panels were also inquired to judge the appropriateness of the question. The success rate was calculated by dividing the number of correct answers by 9. For the appropriateness rating, the panelists were asked to rate it on a 9-point Likert scale from 1 (completely irrelevant) to 9 (extremely relevant), considering the clarity of the clinical presentation, ECG wave quality, and the discrimination of the choices of answers. Different positions of the evaluators (medical students, residents, and specialists, among others) may have different judgments regarding the “Appropriateness” of the ECG questions. However, the goal of this project is to enhance ECG interpretation skills regardless of the position. Hence, it was necessary for each panelist to evaluate the suitability of the question from his or her own perspective to obtain diverse opinions. Using the median and IQR scores calculated from the appropriateness ratings, the validity of the questions was assessed and categorized as follows: “Appropriate,” median 7–9 with an IQR ≤2; “Discussion,” median 4–6 with an IQR ≥2; and “Inappropriate,” median 1–3 with an IQR ≤2.

To reconcile the opinions of professionals in different career stages, we calculated median and IQR as objective indicators of appropriateness. Furthermore, based on the results, the opinions were further reconciled under a moderator control through an active face-to-face discussion in round 2. Round 2 was conducted in two separate online meetings, and panelists who could not attend were invited to watch the meeting recording to summarize all of the expert’s opinions, as in round 0. In round 2, the appropriateness of the ECG questions was finally classified into the following four categories: “Appropriate,” “Candidate,” “Revision,” and “Defer.” Those considered as appropriate for both rounds 1 and 2 were classified as “Appropriate.” Those considered as “ Discussion” in round 1 but appropriate in round 2 were classified as “Candidate.” Those judged as requiring the approval of the nine panelists again to be added to the pool of the ECG questions after some modifications were classified as “Revision.” Those judged to require correction as a *de novo* basis were classified as “Defer.” The categorization of each question was also determined. An ECG test set was finally created by extracting 50 questions from those judged as “Appropriate” or “Candidate,” considering the balance of the categories. The ECG test set will be used in the next phase of our ECG project with a cross-sectional, online assessment.

## Results

As a result of the online test in round 1, the success rate of the nine experts is shown in Table 1. The online tests had no missing data, with all nine experts answering 100 questions. The overall average success rate for the nine experts was 0.89. By category, the success rates were 0.91 for “Ischemia/infarction,” 0.88 for “Rhythms,” 0.92 for “Metabolic/inflammatory,” 0.86 for “Structural,” and 0.88 for “Others.”

**Table 1:** Success rate by category for each of the 9 panelists

		**Success rate by category**					
**Occupation**	**Subspeciality**	Overall	Ischemia/infarction	Rhythms	Metabolic/inflammatory	Structural	Others
Cardiologist	Arrythmia	0.98	0.94	0.98	1.0	1.0	1.0
Cardiologist	Arrythmia	0.90	0.94	0.93	1.0	0.85	0.82
Cardiologist		0.91	0.94	0.95	0.86	0.92	0.82
Cardiologist		0.89	0.94	0.86	0.86	0.92	0.86
Cardiologist	ACHD	0.87	0.78	0.83	1.0	0.85	1.0
Emergency physician		0.71	0.89	0.73	0.57	0.54	0.68
Paramedic	EMT	0.97	1.0	0.96	1.0	0.92	0.95
Clinical laboratory technician		0.91	0.94	0.86	1.0	0.85	0.95
Registered nurse/medical student		0.87	0.78	0.86	1.0	0.92	0.86
**Average score**		0.89	0.91	0.88	0.92	0.86	0.88

*ACHD* Adult congenital heart disease, *EMT* Emergency medical technician

**Fig. 2:**
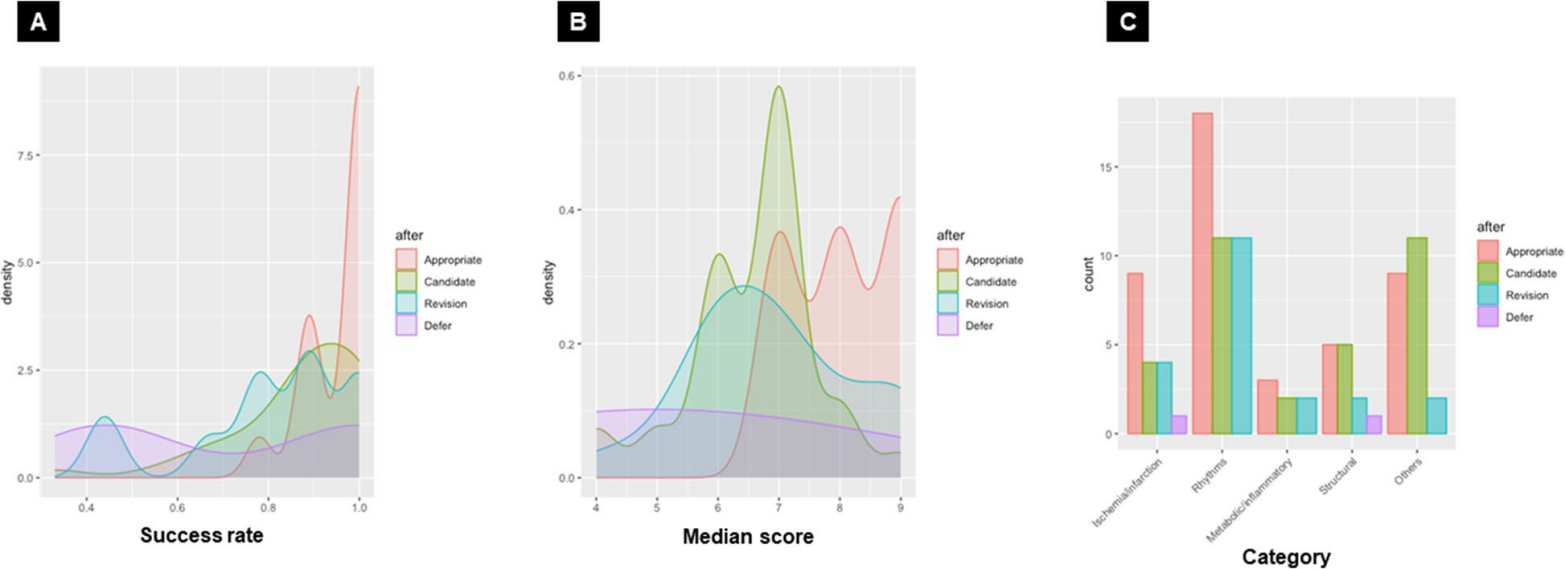
Evaluation of the appropriateness of 100 questions and its relation to success rate, median score, and category. A: difference in the success rate; B: difference in the median score; **C:** difference in the category

In the round 2 results, questions judged as “Appropriate” tended to have a higher success rate and median score of appropriateness than others (Fig. 2A and B). Moreover, questions related to “Ischemia/infarction” and “Rhythm” tended to be judged more appropriately than questions of other categories (Fig. 2C).

**Fig. 3:**
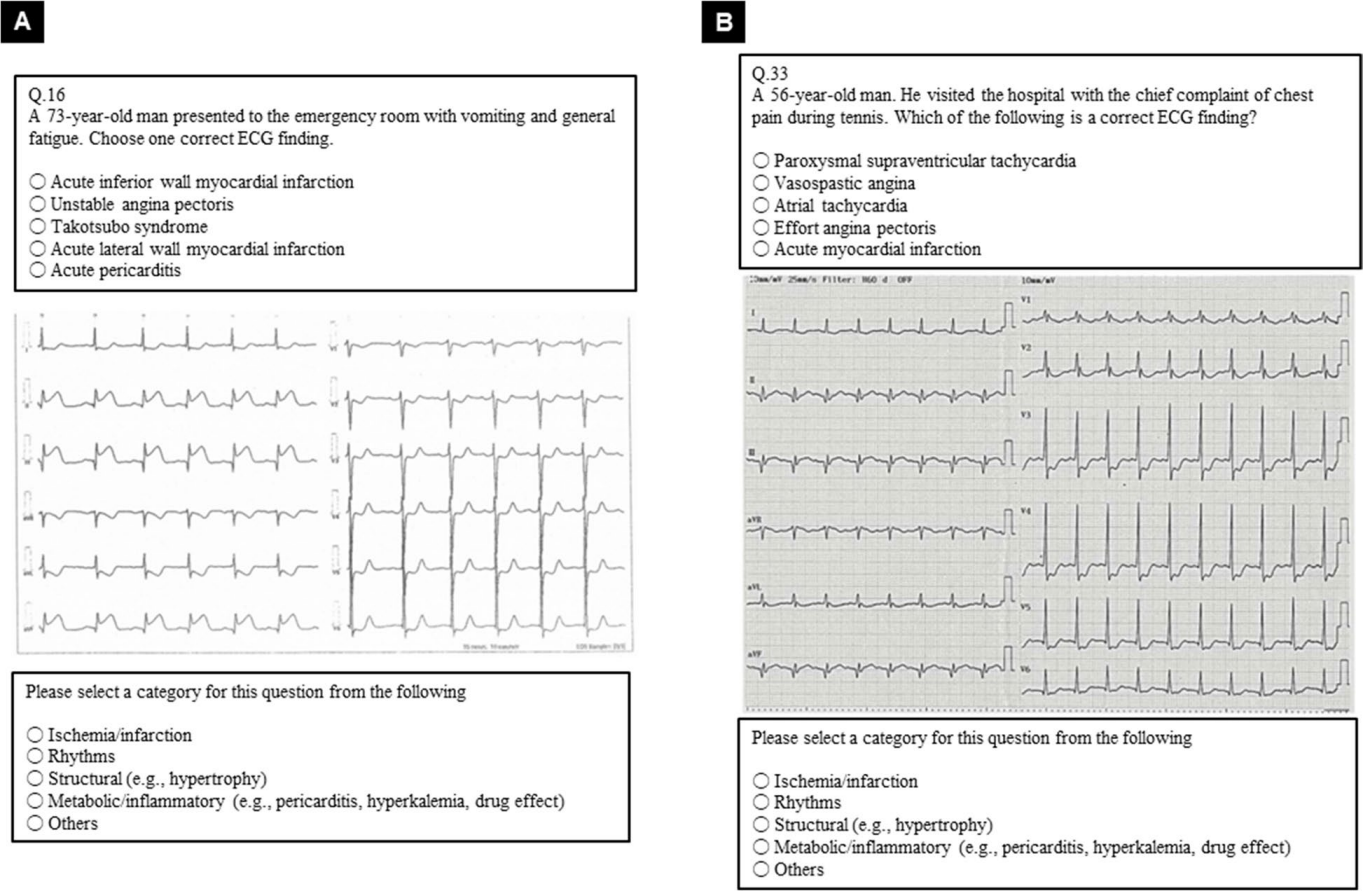
Examples of questions deemed as “Appropriate” and “Revision.”. The ECG questions in the online test are organized as follows: a question text with the patient’s age, sex, and chief complaint; five choices; the ECG waveform in the middle; and five category choices at the bottom. **A** Example of a question deemed as “Appropriate” for both rounds 1 and 2. The category was classified as “Ischemia/infarction” in rounds 1 and 2. In round 1, the median score was high (9) with a low IQR (0); thus, the question was deemed as appropriate, as confirmed in round 2. **B** Example of a question considered as “Revision” after round 2. The median score was as high as 8; however, the IQR was also as high as 4; thus, it was determined as requiring discussion in round 1. The author created the question with the correct answer as effort angina. However, ST-segment elevation in II, III, and aV_F_ was observed in the ECG. After a discussion in round 2, acute myocardial infarction could not be excluded and that the choice and ECG image required modification. ECG = electrocardiogram; IQR = interquartile range

Table 2 shows the appropriateness of the 100 questions before and after a face-to-face expert panel meeting in round 2. In round 1 before round 2, none of the questions were judged as “Inappropriate,” but as many as half of the 100 questions (*n* = 54) were considered as requiring “ Discussion.” The round 2 results revealed that 44% (*n* = 44) were “Appropriate,” 33% (*n* = 33) were “Candidate,” 21% (*n* = 21) were “Revision,” and 2% (*n* = 2) were “Defer” out of a total of 100 questions. Examples of questions that were considered as “Appropriate” and “Revision” via round 1 and 2, respectively, are presented in Fig. 3A and B. Two of the 46 questions deemed “Appropriate” in round 1 were re-evaluated as “Revision” in round 2. Of the 54 questions considered as requiring discussion in round 1, 33 questions were re-evaluated as “Candidate,” 19 as “Revision,” and 2 as “Defer,” respectively. Thus, of the 100-item questions, 44 were considered as “Appropriate” and 33 were considered as “Candidate” for the 77 questions that were candidates for the actual test questions.

**Table 2:** Appropriateness of the 100-item questions before and the after round 2

**Before round 2**	**After round 2**			
	Appropriate	Candidate	Revision	Defer
Appropriate (*n* = 46)	44	0	2	0
Discussion (*n* = 54)	0	33	19	2
Inappropriate (*n* = 0)	0	0	0	0
**Total (*n* = 100)**	44	33	21	2

**Fig 4:**
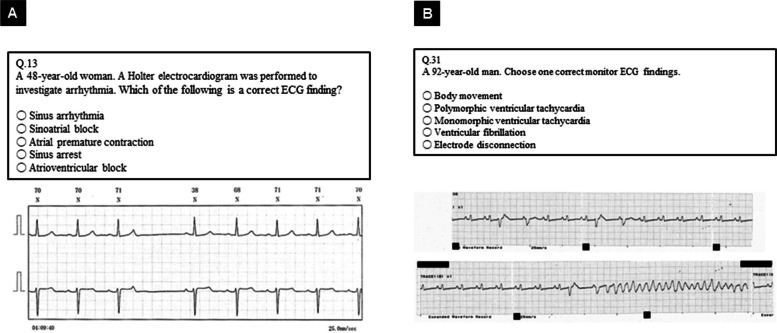
Two questions were deemed as “Appropriate” in round 1 but must be revised in round 2. **A** The success rate was high at 0.89, and the IQR was low at 1.0, indicating that round 1 was classified as “Appropriate.” However, multiple choices could not be excluded, thus requiring modification. Specifically, the AV block could not be excluded as an answer in addition to the contestant’s answer of supraventricular premature contractions. **B** It was deemed appropriate owing to the high median score and low IQR; however, the success rate was low at 0.44. Thus, the reason was verified in round 2. Although the authors had assumed that the answer was ventricular fibrillation, five of the nine experts had answered ventricular tachycardia. They all concurred that it was a clinically important question; however, because the choice could not be excluded, it was decided that a modification was needed, including the ECG imaging. ECG = electrocardiogram; IQR = interquartile range

The detailed rationale for the change in appropriateness after round 2 is presented in Table 3. The most common reason for re-rating from “ Discussion” to “Candidate” was high clinical importance. Additionally, both primary and high-difficulty-level questions were often considered as less appropriate in round 1, and these were re-rated as “Candidate” in round 2. Questions were considered as requiring “Revision” often because of multiple choices that could not be excluded, followed by inappropriate descriptions in the choices or questions. Two questions that were reclassified as “Revision” in round 2, although they were considered “Appropriate” in round 1, are presented in Fig. 4.

**Table 3:** Reasons for change in appropriateness after round 2

**Reasons**	**Changes in appropriateness**			
	Appropriate→Revision (*n* = 2/46)	Discussion→Candidate (*n* = 33/54)	Discussion→Revision (*n* = 19/54)	Discussion→Defer (*n* = 2/54)
**Clinical importance**				
High clinical importance		29		
Low clinical importance				1
**Difficulty**				
High difficulty level question		9		
Basic level question		13		
**Question**				
Multiple choices that cannot be excluded	2		11	
Inappropriate description of the choice			6	1
Inappropriate description in the question	1		5	
Inappropriate image			4	

^a^There are overlapping reasons

## Discussion

In the current study, the following observations were made: (1) Approximately half of the questions were considered as requiring discussion in round 1; (2) in round 1, high- and low-difficulty questions having a lower rating of appropriateness were often not judged as “Appropriate”; (3) in round 2, various reasons were determined for the decision as “Revision”; however, the most common rationale was the presence of multiple choices that could not be excluded; (4) although two skilled cardiologists prepared the 100 ECG questions, 23% of the questions were ultimately determined as requiring modification. From the 77 ECG questions that were ultimately determined to be “Appropriate” or “Candidate,” a valid test set consisting of 50 questions was successfully created.

### Impact of the RAM on validating MCQs

Prior research on MCQs has shown that some item-writing flaw, such as vague terms and no correct or more than one correct answer, arises even when the writers of the questions are specialists [16]. Balaha et al. reviewed previous papers on MCQs, noting that most reports found item-writing flaws in more than 60% of the test questions. However, our study found item-writing flaws in 23% of the questions, which is of lower frequency than in previous studies. The frequency of item-writing flaws varies widely across MCQ resources; review-related MCQ books and online-shared test banks demonstrated low-quality questions [12]. In our study, two cardiologists skilled in ECG training created the candidate 100-item questions, which may have influenced the optimal results.

However, the most important finding from this study is that even questions created by skilled experts had item-writing flaws in approximately a fourth of questions. Flawed questions benefit borderline students and, conversely, adversely affect high-achieving students [17]. Thus, avoiding flawed questions for a fair assessment is crucial. Flawed questions should be deleted or corrected and revalidated, and in this study, RAM was useful for this validation. The Certified Cardiovascular Disease Specialist Examination produced by the American Board of Internal Medicine consists of MCQs in question format, and one of the examination items includes ECG decoding. RAM might be used to create test sets with even higher validity although a committee of medical specialists has been established to prepare and review the examination questions.

### Potential indicators of appropriateness: median score, IQR, and success rate

In this study, approximately half (54%) of the item-questions were automatically deemed as requiring discussion using a median score and IQR in round 1. Meanwhile, most of the questions (95.7%) categorized as “Appropriate” in round 1 were considered as “Appropriate” even after round 2. The median score and IQR, used for the first time in this study to identify the appropriateness of ECG questions, were helpful in screening for round 2 as a novel quantitative indicator.

In this study, of the 54 questions that required discussion in round 1, 33 (61.1%) were classified as “Candidate” questions in round 2. Even if all experts can answer a question correctly, questions with a high degree of difficulty tend to have low appropriateness ratings in round 1. Although this study used a median score and IQR to determine appropriateness, questions that were ultimately judged as appropriate also tended to have higher success rates. Hence, adding the success rate to these indicators may improve screening accuracy in round 1. Further verification of the utility of these indicators is warranted.

### Appropriate number of panelists and potential role of multidisciplinary expert panels

There is limited knowledge on the adequate number of panelists in considering the validation of MCQs [12, 16–18]. According to the RAM User’s Manual, the recommended number of panelists is 7–15 for a panel discussion [14]. This number allows everyone’s participation in the discussion and ensures diversity. In the medical discipline, several previous studies have examined MCQs using consensus-building methods other than RAM. Nevertheless, most had fewer than five panelists, less than the number recommended in RAM [17, 18]. In such cases, there is concern that diversity may be compromised. Because this study involved consensus building by nine panelists, more reliable validation of the RAM might have been obtained. Having an odd number of experts might be helpful in avoiding ties in cases of competitive selection. Although diversity is expected to increase with the number of participants, it may be difficult for all to participate. In recent years, an increase in web-based panel meetings have been observed in consensus-building methods using RAM [19]. Therefore, we addressed these issues by conducting an online meeting so that remote panelists could participate and allow those who could not attend to view a video recording of the meeting.

Moreover, diversity of occupations is also essential for aggregating diverse opinions. The RAM User’s Manual recommends that panelists include a wide range of professions related to the topic of discussion [14]. Jansen et al. examined the creation of a consensus-based “license to prescribe” exam question for medical students through 10 panelists [16]. However, the panelists lacked diversity, consisting only of physicians or pharmacists specializing in clinical pharmacology. In the studies by Barlow et al. and Tarrant et al., the number of panelists was small (less than five) and diversity was limited, with panels consisting of two areas of expertise [17, 18]. In this study, the nine panelists comprised cardiologists, an emergency physician, and multiple professionals (emergency medical technician, clinical laboratory technician, and nurse) who handle the ECG as a specialist or in daily practice according to the RAM recommendations. In our study, the diversity of these occupations may have helped provide a more accurate discussion, resulting in a lower item-writing flaws rate compared with those of previous reports. However, the selection of appropriate occupations and the number of occupations constituting the expert panel has not been determined and needs to be verified in the future.

### Limitations

This study has several limitations. First, no statistical analysis was performed for the following two reasons: (1) The number of item-questions verified is small, and (2) given this is the first study to use RAM for MCQ validation and there are few previous studies for comparison, the emphasis of this study is to be descriptive. However, a further large-scale test will be administered to 500 general respondents in the future, using the 50 validated questions generated in this study. The actual validity of the questions will be verified in detail there. Second, the current study only focused on ECGs, and it is not clear whether our results can be employed to other fields. However, because MCQs are increasingly incorporated into medical education in various disciplines, more opportunities will be available to validate them using this system. Hence, we hope that validation will also be conducted in other fields. Third, a conclusion on the appropriate number of expert panelists and occupations for consensus building to prepare MCQs has yet to be attained. No reports have emphasized on this issue; therefore, in this study, no comparative verification has been conducted. For the first time, we organized an expert committee according to the RAM for the preparation of the MCQs and were able to validate it step-by-step without complications. A comparison with the results of our study will likely provide guidelines for the appropriate organization of expert committees in the future.

## Conclusions

This study shows that the RAM might be useful in validating MCQs. Simple indicators of appropriateness using the median score and IQR might be helpful in efficiently extracting appropriate item-questions. Additionally, flawed questions tend to have specific patterns, and it may be advisable to consider these tendencies while building consensus. Notably, although two skilled cardiologists determined that the questions were appropriate, approximately a fourth of the questions were identified by the RAM as inappropriate. Using the RAM to analyze diverse opinions and build consensus may help create higher-quality tests that are more effective for learning.

## Data Availability

The datasets used and/or analyzed during the current study are available from the corresponding author on reasonable request.

## References

[1] Rubinstein J, Dhoble A, Ferenchick G (2009). Puzzle based teaching versus traditional instruction in electrocardiogram interpretation for medical students–A pilot study. BMC Med Educ.

[2] Fibrinolytic Therapy Trialists’ (FTT) Collaborative Group. Indications for fibrinolytic therapy in suspected acute myocardial infarction: Collaborative overview of early mortality and major morbidity results from all randomised trials of more than 1000 patients. Lancet. 1994; 343(8893): 311–22. 10.1016/S0140-6736(94)91161-4. 7905143

[3] Boersma E, Maas AC, Deckers JW, Simoons ML (1996). Early thrombolytic treatment in acute myocardial infarction: reappraisal of the golden hour. Lancet.

[4] Viljoen CA, Scott Millar R, Engel ME, Shelton M, Burch V (2019). Is computer-assisted instruction more effective than other educational methods in achieving ECG competence amongst medical students and residents? A systematic review and meta-analysis. BMJ Open.

[5] McAloon C, Leach H, Gill S, Aluwalia A, Trevelyan J (2014). Improving ECG competence in medical trainees in a UK district general hospital. Cardiol Res.

[6] Hurst JW (2000). Methods used to interpret the 12-lead electrocardiogram: Pattern memorization versus the use of vector concepts. Clin Cardiol.

[7] Kopeć G, Magoń W, Hołda M, Podolec P (2015). Competency in ECG interpretation among medical students. Med Sci Monit.

[8] Cook DA, Oh SY, Pusic MV (2020). Accuracy of physicians’ electrocardiogram interpretations: a systematic review and meta-analysis. JAMA Intern Med.

[9] Cook DA, Oh SY, Pusic MV (2022). Assessments of physicians’ electrocardiogram interpretation skill: a systematic review. Acad Med.

[10] Brunnquell A, Degirmenci U, Kreil S, Kornhuber J, Weih M (2011). Web-based application to eliminate five contraindicated multiple-choice question practices. Eval Health Prof.

[11] Downing SM. Construct-irrelevant variance and flawed test questions: Do multiple-choice item-writing principles make any difference? Acad Med. 2002; 77(10); Suppl: S103–S104. 10.1097/00001888-200210001-00032. 10.1097/00001888-200210001-00032 12377719

[12] Balaha MH, El-Ibiary MT, El-Dorf AA, El-Shewaikh SL, Balaha HM (2022). Construction and writing flaws of the multiple-choice questions in the published test banks of obstetrics and gynecology: adoption, caution, or mitigation?. Avicenna J Med.

[13] Rodríguez-Díez MC, Alegre M, Díez N, Arbea L, Ferrer M (2016). Technical flaws in multiple-choice questions in the access exam to medical specialties (‘examen MIR’) in Spain (2009–2013). BMC Med Educ.

[14] Fitch K, Bernstein SJ, Aguilar MD, Burnand B, LaCalle JR, Lázaro P, van Het Loo M, McDonnell J, Vader JP, Kahan JP. The rand/UCLA appropriateness method user’s manual. Rand Publishing. 2001. https://www.rand.org/content/dam/rand/pubs/monograph_reports/2011/MR1269.pdf.

[15] Inaba S, Yamamoto K, Kaga T, Wannous M, Sakata M, Yamaguchi O, Furukawa TA (2023). Protocol for development of an assessment tool for competency of ECG interpretation: Expert consensus by the rand/UCLA appropriateness method and cross-sectional testing using multidimensional item response theory. BMJ Open.

[16] Jansen DRM, Keijsers CJPW, Kornelissen MO, Olde Rikkert MGM, Kramers C, (on behalf of the education working group of the Dutch Society for Clinical Pharmacology and Biopharmacy). Towards a “prescribing license” for medical students: Development and quality evaluation of an assessment for safe prescribing. Eur J Clin Pharmacol. 2019; 75(9): 1261–8. 10.1007/s00228-019-02686-1. 10.1007/s00228-019-02686-1 31104076

[17] Tarrant M, Ware J (2008). Impact of item-writing flaws in multiple-choice questions on student achievement in high-stakes nursing assessments. Med Educ.

[18] Barlow PB, Skolits G, Heidel RE, Metheny W, Smith TL (2015). Development of the biostatistics and clinical epidemiology skills (BACES) assessment for medical residents. Postgrad Med J.

[19] Sparks JB, Klamerus ML, Caverly TJ, Skurla SE, Hofer TP, Kerr EA, Bernstein SJ, Damschroder LJ (2022). Planning and reporting effective web-based rand/UCLA appropriateness method panels: Literature review and preliminary recommendations. J Med Internet Res.

